# HSP90 interacts with VP37 to facilitate the cell-to-cell movement of broad bean wilt virus 2

**DOI:** 10.1128/mbio.02500-24

**Published:** 2025-02-19

**Authors:** Myung-Hwi Kim, Seok-Yeong Jang, Ji-Soo Choi, Sora Kim, Yubin Lee, Suejin Park, Sun-Jung Kwon, Jang-Kyun Seo

**Affiliations:** 1 Department of Agricultural Biotechnology, Seoul National University, Seoul, South Korea; 2 Department of International Agricultural Technology, Seoul National University, Pyeongchang, South Korea; 3 Department of Horticulture, Jeonbuk National University, Jeonju, South Korea; 4 Institutes of Green Bio Science and Technology, Seoul National University, Pyeongchang, South Korea; University of California at Riverside, Riverside, California, USA; Agriculture and Agri-Food Canada, London, Ontario, Canada

**Keywords:** BBWV2, VP37, HSP90, cell-to-cell movement, tubule formation

## Abstract

The systemic spread of viruses in plants requires successful viral cell-to-cell movement through plasmodesmata (PD). Viral movement proteins (MPs) interact with cellular proteins to modify and utilize host transport routes. Broad bean wilt virus 2 (BBWV2) moves from cell to cell as a virion through the PD gated by VP37, the MP of BBWV2. However, the host proteins that function in the cell-to-cell movement of BBWV2 remain unclear. In this study, we identified cellular heat shock protein 90 (HSP90) as an interacting partner of VP37. The interaction between HSP90 and VP37 was assessed using the yeast two-hybrid assay, co-immunoprecipitation, and bimolecular fluorescence complementation. Tobacco rattle virus-based virus-induced gene silencing analysis revealed that *HSP90* silencing significantly inhibited the systemic spread of BBWV2 in *Nicotiana benthamiana* plants. Furthermore, *in planta* treatment with geldanamycin (GDA), an inhibitor of the chaperone function of HSP90, demonstrated the necessity of HSP90 in successful cell-to-cell movement and systemic infection of BBWV2. Interestingly, GDA treatment inhibited the HSP90-VP37 interaction at the PD, resulting in the inhibition of VP37-derived tubule formation through the PD. Our results suggest that the HSP90-VP37 interaction regulates VP37-derived tubule formation through the PD, thereby facilitating the cell-to-cell movement of BBWV2.

**IMPORTANCE:**

This study highlights the regulatory role of heat shock protein 90 (HSP90) in facilitating the cell-to-cell movement of broad bean wilt virus 2 (BBWV2). HSP90 interacted with VP37, the movement protein of BBWV2, specifically at plasmodesmata (PD). This study demonstrated that the HSP90-VP37 interaction is crucial for viral cell-to-cell movement and the formation of VP37-derived tubules, which are essential structures for virus transport through the PD. The ATP-dependent chaperone activity of HSP90 is integral to this interaction, as demonstrated by the inhibition of virus movement upon treatment with geldanamycin, which disrupts the function of HSP90. These findings elucidate the molecular mechanisms underlying the cell-to-cell movement of plant viruses and highlight the role of HSP90 in viral infection. This study suggests that the chaperone activity of HSP90 may function in changing the conformational structure of VP37, thereby facilitating the assembly and function of virus-induced structures required for viral cell-to-cell movement.

## INTRODUCTION

Plant viruses are obligate intracellular parasites that depend on living host cells for proliferation. They induce varying degrees of biotic stress by hijacking host cellular machinery and disturbing metabolic functions during infection, resulting in physiological and morphological changes in host plants (1–3). In addition, infection involves multiple compatible interactions between viral and host proteins at various stages of the viral life cycle, including gene expression, replication, and spread (4–6). Thus, interactions between viral and host proteins have markedly influenced the evolution of plant viruses (2).

Viral transport within plants relies on non-structural proteins known as movement proteins (MPs). In most plant viruses, MPs orchestrate intracellular, intercellular, and long-distance movement to facilitate systemic infection (7). Although viral MPs are structurally diverse, they share common characteristics, such as localization to plasmodesmata (PD), PD gating, and interaction with host proteins during these processes (8, 9). The reliance of viral movement on host proteins is strongly associated with the physical interactions between viral MPs and host proteins. Therefore, identifying host proteins that interact with viral MPs is crucial for elucidating the molecular mechanisms underlying the intracellular and intercellular movements of plant viruses. Accumulating evidence reveals that viral MPs interact with various host proteins associated with PD and cellular transport systems to modify and utilize existing transport routes in plants (6, 10, 11).

Broad bean wilt virus 2 (BBWV2), a member of the genus *Fabavirus* in the family *Secoviridae*, is an emerging viral pathogen with a broad host range. It poses a significant threat to various economically important crops, including pepper and legumes (12). The BBWV2 genome consists of two single-stranded positive-sense RNAs (RNA1 and RNA2) that are approximately 5,960 and 3,600 nucleotides long, respectively (13). RNA1 contains a single open reading frame that encodes one polyprotein precursor. This precursor is proteolytically cleaved to produce five mature proteins: the protease cofactor, nucleoside triphosphate (NTP)-binding motif, viral genome-linked protein, protease, and RNA-dependent RNA polymerase. RNA2 encodes two largely overlapping polyproteins using two alternative in-frame start codons. These polyproteins differ only at their N-termini and are proteolytically cleaved at the identical cleavage sites to produce four mature proteins: a replication-associated protein of 53 kDa (VP53), a viral movement protein of 37 kDa (VP37), as well as large and small coat proteins (13).

The role of VP37 in viral infection has been extensively demonstrated (13–16). VP37 is localized to the PD and forms tubule structures that facilitate the tubule-guided cell-to-cell movement of BBWV2 virions (13, 16). In addition, it can bind single-stranded nucleic acids and suppress RNA silencing (17). However, the host cellular proteins that interact with VP37 are yet to be identified. In our previous study, we engineered an infectious cDNA construct of BBWV2 RNA2 to express VP37 tagged with a Flag epitope (Asp-Tyr-Lys-Asp-Asp-Asp-Asp-Asp-Lys) at the C-terminus (VP37-Flag) during BBWV2 replication (13, 18). We also demonstrated that tagging VP37 with a Flag epitope at the C-terminus did not impair VP37 function in viral infectivity (13). Therefore, the present study aimed to identify the host cellular proteins that interact with VP37 using this Flag-tagged BBWV2 construct in combination with immunoprecipitation followed by liquid chromatography-tandem mass spectrometry (LC-MS/MS). Based on this approach and additional experiments, we identified cellular heat shock protein 90 (HSP90) as an interacting partner of VP37 in *Nicotiana benthamiana*. Our study also revealed that the HSP90-VP37 interaction occurred at the PD and was essential for the cell-to-cell movement of BBWV2, suggesting that HSP90 is a host factor that regulates BBWV2 movement through its interaction with VP37.

## MATERIALS AND METHODS

### Plant growth and agroinfiltration

*N. benthamiana* plants were grown in an insect-free growth chamber under controlled conditions, with a 16 h light period at 26°C followed by an 8 h dark period at 24°C. For agroinfiltration, T-DNA-based binary vector constructs were transformed into *Agrobacterium* strain EHA105. Next, the *Agrobacterium* cultures carrying each construct were grown overnight at 30°C in a yeast extract-peptone (YEP) medium supplemented with kanamycin (100 µg/mL) and acetosyringon (20 µM). The cultures were then centrifugated at 3,000 rpm for 10 min, and the resulting bacterial pellet was resuspended to 0.5 absorbance at 600 nm (OD_600_) in an infiltration buffer [Murashige-Skoog (MS) salts, 10 mM 2-(N-morpholino)ethanesulfonic acid (MES), pH 5.6, 200 µM acetosyringon]. The resuspended Agrobacteria were incubated at 30°C for 4 h and infiltrated into the abaxial surface of *N. benthamiana* leaves using a 1 mL syringe. Each inoculation experiment was repeated independently at least thrice, with at least three plants per construct in each experiment.

### Immunoprecipitation and liquid chromatography coupled with tandem mass spectrometry analysis

*N. benthamiana* plants were infiltrated with a mixture of *Agrobacterium* cultures containing either pBBWV2-RP1-R1 + pBBWV2-RP1-R2 (this combination was designated as BBWV2-RP1) or pBBWV2-RP1-R1 + pBBWV2-R2-53/37-Flag (this combination was designated as BBWV2-53/37-Flag) as previously described (13). At 14 days post-infiltration (dpi), crude extracts were obtained from systemic leaves of healthy plants, BBWV2-RP1-infected plants, or BBWV2-53/37-Flag-infected plants by homogenizing the leaves in three volumes of protein extraction buffer (20 mM Tris–HCl at pH 7.5, 300 mM NaCl, 5 mM MgCl_2_, 5 mM dithiothreitol, 0.5% Triton X-100, proteinase inhibitor cocktail [Sigma, USA]). Cell debris was removed via centrifugation at 18,000 × *g* for 20 min at 4°C. The resulting supernatants were then incubated with anti-Flag antibody-conjugated magnetic beads (Thermo Fisher Scientific Inc., USA) for 16 h at 4°C. After incubation, the immunocomplexes were washed five times with 1.5 mL of the protein extraction buffer. The samples were analyzed using 10% sodium dodecyl sulfate-polyacrylamide gel electrophoresis (SDS-PAGE) and stained with Coomassie blue; the Xpert prestained protein marker (GenDEPOT, USA) was used as a molecular weight marker. The bands of interest were excised from the gel for in-gel trypsin digestion followed by LC-MS/MS analysis as described previously (19). LC-MS/MS analysis was conducted at the Yonsei Proteome Research Center (Seoul, South Korea). Xcalibur (Thermo Fisher Scientific Inc., USA) was used to generate the peak lists. The data were identified by searching the National Center for Biotechnology Information database using the MASCOT search engine (http://www.matrixscience.com, Matrix Science, USA).

### Yeast two-hybrid assay

Yeast two-hybrid assay (YTHA) was performed using the Matchmaker Two-Hybrid System 2 (Clontech, USA) as described previously (20). The coding sequences of VP37, VP53, HSP90-1, HSP90-2, and HSP90-3 were amplified using Q5 High-Fidelity DNA polymerase (NEB, USA) and the appropriate primer pairs (Table S1). HSP90-1, HSP90-2, and HSP90-3 were fused downstream of GAL4-AD through in-frame insertion at the *Sma*I and *Xho*I sites of the pGADT7 vector. VP37 and VP53 were fused downstream of GAL4-BD via in-frame insertion using the *EcoR*I and *BamH*I sites of the pGBKT7 vector. The sequences of all constructed clones were validated using DNA sequencing. Yeast competent cells (AH109) were co-transformed with pGADT7 and pGBKT derivatives using the lithium acetate method (21). They were then selected on SD/-Leu/-Trp (SD/-LW), SD/-Leu/-Trp/-His (SD/-LWH), or SD/-Leu/-Trp/-His/-Ade (SD/-LWHA) media. The interaction between the SV40 large T antigen_(84-708)_ and murine p53_(72-390)_ was used as a positive control, whereas the interaction with human lamin C_(66-230)_ served as a negative control. All protein-protein interactions were validated by conducting α-galactosidase activity assays using the X-α-Gal reagent (Clontech, USA).

### Co-immunoprecipitation and western blot analysis

DNA templates for T7 *in vitro* transcription were prepared via PCR amplification using specific primers and templates (Table S1): HSP90-T7-Fw and 3E-PolyA-Rv for HSP90-2-Flag and VP37-T7-Fw and 3E-PolyA-Rv for VP37-Myc. The resulting PCR products were purified using the Geneclean Turbo kit and subjected to *in vitro* coupled transcription/translation reactions using the TNT Quick Coupled Transcription/Translation System (Promega, USA) according to the manufacturer’s instructions. The reaction products (i.e., HSP90-2-Flag and VP37-Myc) were mixed accordingly for co-immunoprecipitation (Co-IP) and subsequently incubated with anti-Flag antibody-conjugated magnetic beads (Thermo Fisher Scientific Inc., USA) for 1 h at 26°C. Next, the immunocomplexes were washed five times with 1.5 mL of protein extraction buffer and subjected to western blot analysis. The resulting samples were separated through 12% SDS-PAGE and transferred onto a polyvinylidene difluoride membrane. The membrane was probed with either anti-Flag (Clontech, Japan) or anti-Myc (Santa Cruz Biotechnology, USA) primary antibodies. A secondary antibody conjugated to horseradish peroxidase (Cell Signaling Technology, USA) was used to visualize the antigens through an ECL western blotting detection system (GE Healthcare Life Sciences, USA).

### Bimolecular fluorescence complementation assay, ectopic expression of fluorescently tagged proteins, and cellular fluorescence imaging

The coding sequences of VP37, VP53, and HSP90 were PCR-amplified from the clones used in YTHA using specific primer pairs (Table S1) and inserted into the pENTR1A vector (Invitrogen, USA) using the *Sal*I and *Xho*I sites. The resulting clones, pENTR1A-VP37, pENTR1A-VP53, and pENTR1A-HSP90, were then used to subclone VP37, VP53, and HSP90 into the bimolecular fluorescence complementation (BiFC) vector pSAT4-DEST-nEYFP-C1 (containing the N-terminal half of YFP, comprising amino acids 1–174 [nYFP]) and pSAT5-DEST-cEYFP-C1 (containing the C-terminal half of YFP, comprising amino acids 175–239 [cYFP]), respectively, using the LR Clonase II Enzyme mix (Invitrogen, USA). The resulting constructs were named nYFP-VP37, nYFP-VP53, and cYFP-HSP90, respectively. The sequences of all constructed clones were validated through DNA sequencing. PZP-YFP was used to express free YFP upon agroinfiltration (22). PZP-ER-CFP, which expresses CFP fused with the endoplasmic reticulum (ER)-targeting signal and ER retention sequence (KDEL) (ER-CFP), was used to express an ER marker (22). To stain the PD, *N. benthamiana* leaves were infiltrated with a solution of 0.1 mg/mL aniline blue (Sigma-Aldrich, USA) and incubated for 30 min (13). PZP-VP37-GFP was used to examine the subcellular localization of VP37 (13). The coding sequence of HSP90-2 was PCR-amplified using the appropriate primers (Table S1). Next, it was inserted in-frame upstream of the GFP gene in the PZP-GFP vector using the *Stu*I and *Spe*I sites (13). The resulting construct, PZP-HSP90-2-GFP, was used to examine the subcellular localization of HSP90-2. Plasmid DNA of the binary vector constructs, including the BiFC clones, was transformed into the *Agrobacterium* strain EHA105 and delivered to *N. benthamiana* leaves through agroinfiltration (22). Cellular fluorescence signals in plant leaves agroinfiltrated with BiFC and other constructs expressing fluorescent proteins were observed at 3 dpi using a Leica SP8 laser-scanning confocal microscope (Leica, Germany) equipped with specific laser/filter combinations for yellow fluorescent protein (YFP; excitation at 514 nm), red fluorescent protein (RFP; excitation at 594 nm), green fluorescent protein (GFP; excitation at 488 nm), and chloroplasts (excitation at 568 nm).

### Viral sources, RNA extraction, and quantification

Full-length cDNA clones of BBWV2-RP1 and its derivative constructs were generated in previous studies (18, 23, 24). We used these viral sources and inoculated them into the leaves of 2-week-old *N. benthamiana* plants via agroinfiltration. The combination of pBBWV2-RP1-R1 and pBBWV2-R2-GFP was designated as BBWV2-GFP (25). The β-glucuronidase (GUS) gene was PCR-amplified using a specific primer pair (Table S1). It was then cloned into pBBWV2-R2-OE (18) using the *Bgl*II and *Avr*II sites to generate pBBWV2-R2-GUS. The combination of pBBWV2-RP1-R1 and pBBWV2-R2-GUS was designated as BBWV2-GUS. RNA was extracted using the Hybrid-R RNA extraction kit (GeneAll, South Korea) according to the manufacturer’s instructions. To confirm the systemic infection of plants inoculated with BBWV2, total RNA was isolated from the upper non-inoculated leaves and subjected to RT-PCR using a BBWV2-specific primer pair (5′-CAGAGAAGTGGTTGGTCCCGTG-3′ and 5′-ATGGGAGGCTAGTGACCTACG-3′) (13). To quantify the accumulation levels of *HSP90-2* and *HSP90-3* mRNAs, RT followed by quantitative PCR (RT-qPCR) was performed using the diastar onestep multiplex qRT-PCR kit (Solgent, South Korea) and Bio-Rad CFX Maestro 2.3 system (Bio-Rad, USA) with the following specific primers: qHSP90-124-Fw (5′-CGCTAGACAAGATCCGCTTTG-3′) and qHSP90-310-Rv (5′-CCTTGGTCCCTGACCTAGCA-3′) for the detection of *HSP90-2* and *HSP90-3* (Fig. S1); Nb-actin-qRT-Fw (5′-CGAGGAGCATCCAGTCCTCT-3′) and Nb-actin-qRT-Rv (5′-GTGGCTGACACCATCACCAG-3′) for actin detection. The actin gene served as an internal reference to normalize the RT-qPCR results. Three biological replicates and five technical replicates were analyzed using RT-qPCR for each sample.

### Measurement of chlorophyll contents

The relative chlorophyll content in healthy or BBWV2-infected *N. benthamiana* leaves was measured using the SPAD 502 plus chlorophyll meter (Konica Minolta, Japan) according to the manufacturer’s instructions. The SPAD readings were obtained from three different areas of each leaf, and the experiment was performed thrice with three plants in each group.

### Tobacco rattle virus-based virus-induced gene silencing

A 300 bp partial fragment of *HSP90-2* was PCR-amplified using a specific primer pair (Table S1; Fig. S1). Next, it was cloned into pTRV2 (26) using the *Sal*I site to generate pTRV2-HSP90. pTRV-GUS, which contains a partial GUS sequence was used as a negative control (27). Tobacco rattle virus (TRV)-based virus-induced gene silencing (VIGS) constructs were agroinfiltrated into the abaxial leaf surface of 2-week-old *N. benthamiana* plants.

### Imaging of fluorescence in living plants

GFP fluorescence signals in living plants were visualized using a FOBI fluorescence imaging system (NeoScience, Korea). The system was equipped with a blue light source for excitation at 470 nm and an emission filter (530 nm short-pass) that effectively removed autofluorescence signals from chlorophyll (25).

### Geldanamycin treatment

Geldanamycin (GDA) solutions were prepared at concentrations of 0, 1, and 10 µM in 1% dimethyl sulfoxide (28). GDA solutions at each concentration were syringe-infiltrated into the leaf areas previously agroinfiltrated with BiFC constructs (i.e., nYFP-VP37 + cYFP-HSP90-2), BBWV2-GFP, BBWV2-GUS, or PZP-VP37-GFP.

### Histochemical GUS assay

GUS expression driven by BBWV2-GUS infection was assessed using a histochemical GUS assay as previously described (29). Briefly, *N. benthamiana* leaves agroinfiltrated with BBWV2-GUS (0.005 OD_600_) were obtained at 5 dpi. Next, they were vacuum-infiltrated with the colorimetric GUS substrate 5-bromo-4-chloro-3-indoyl β-D-glucuronic acid, cyclohexylammonium salt (X-gluc) (1.2 mM) dissolved in a buffer containing 0.5 mM potassium ferricyanide, 0.5 mM potassium ferrocyanide, and 10 mM EDTA. After 12 h of incubation at 25°C, the leaves were bleached in 70% ethanol and examined using a dissecting microscope to measure the diameters of the GUS foci.

### Reproducibility and statistical analyses

All experimental results were obtained from three independent experiments, with each experiment involving at least three plants per condition. Statistical significance between the experimental groups was evaluated using one-way analysis of variance with Tukey’s honestly significant difference (HSD) test (*P* < 0.05) or a paired Student’s *t*-test (*P < 0.05, **P < 0.01). Statistical data are presented as the mean ± standard deviation (SD) from three replicates, with each group comprising at least nine plants.

## RESULTS

### HSP90 is a cellular interacting partner of VP37

**Fig 1:**
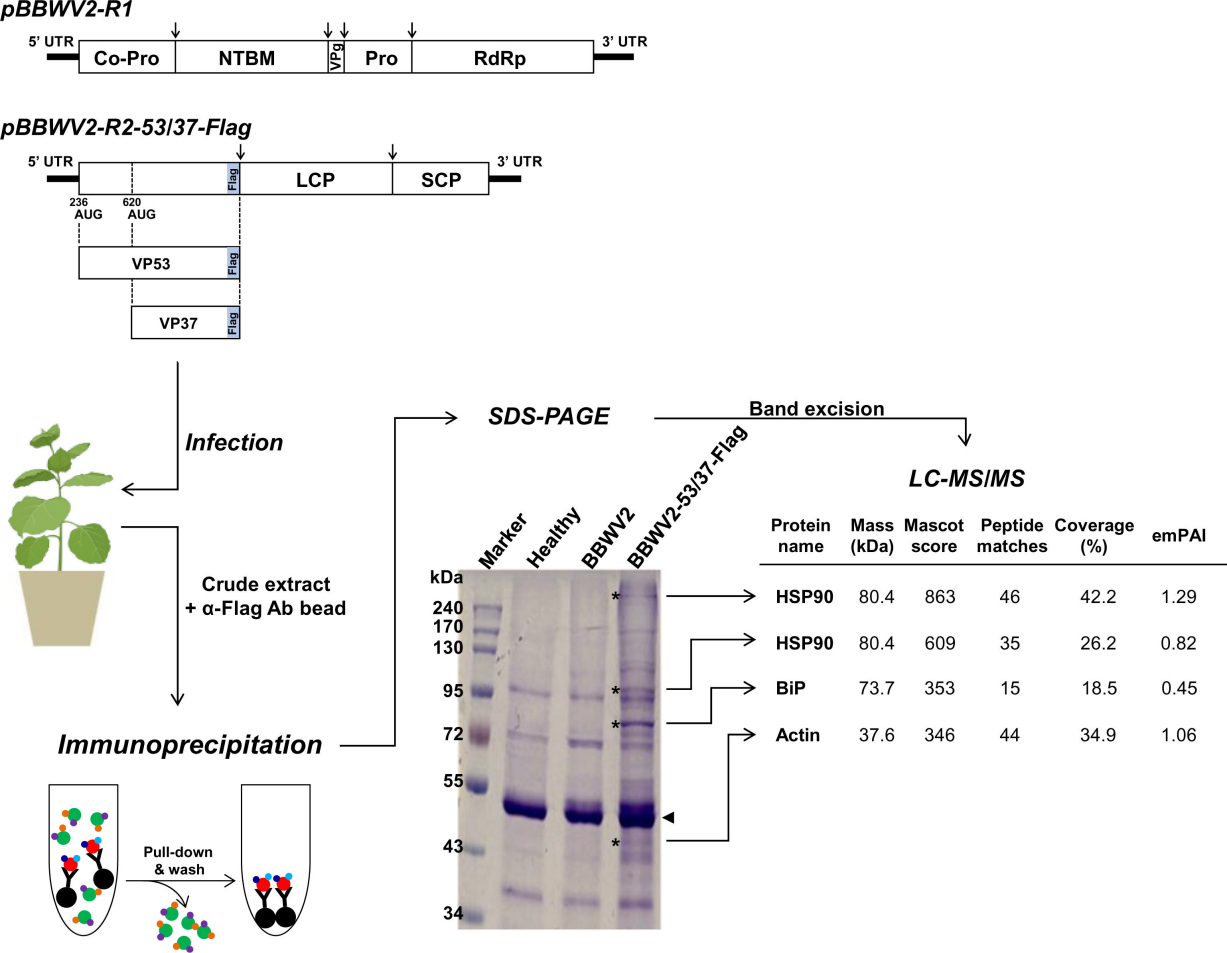
Workflow for the identification of cellular interacting protein partners of VP37 and VP53 under broad bean wilt virus 2 (BBWV2) infection conditions. A schematic representation of the BBWV2 recombinant constructs shows the in-frame fusion of a Flag-tag at the C-terminus of VP37/VP53, thereby expressing VP37-Flag and VP53-Flag during viral replication. *Nicotiana benthamiana* plants were agroinfiltrated with BBWV2-53/37-Flag (a mixture of *Agrobacterium* cultures containing pBBWV2-RP1-R1 + pBBWV2-R2-53/37-Flag). At 14 dpi, crude extracts were prepared by homogenizing the upper systemically infected leaves and subjected to immunoprecipitation using magnetic beads conjugated with anti-Flag antibodies. Crude extracts prepared from systemic leaves of healthy plants or BBWV2-RP1-infected plants were used as controls in the immunoprecipitation experiments. The resulting products were analyzed using SDS-PAGE, and the bands of interest (marked with asterisks) were analyzed through LC-MS/MS. The identified proteins and their MS/MS spectral data are presented.

To identify host interacting partner proteins of VP37 and VP53, we expressed VP37-Flag and VP53-Flag in *N. benthamiana* plants using the modified infectious cDNA constructs of BBWV2 (pBBWV2-RP1-R1 and pBBWV2-R2-53/37-Flag; this combination was designated as BBWV2-53/37-Flag) (Fig. 1) (13). BBWV2-53/37-Flag contains VP53 and VP37 cistrons fused in-frame with Flag, thereby expressing both VP37-Flag and VP53-Flag during viral replication (13). *N. benthamiana* plants were agroinfiltrated with either BBWV2-53/37-Flag or BBWV2-RP1. At 14 dpi, crude plant extracts were obtained by homogenizing the upper symptomatic leaves infected with BBWV2-53/37-Flag. The crude extracts were subsequently subjected to immunoprecipitation using anti-Flag antibody-conjugated magnetic beads. In parallel, crude extracts were similarly prepared from systemic leaves of healthy plants and BBWV2-infected plants, serving as controls in the immunoprecipitation experiments. The resulting products were analyzed using SDS-PAGE followed by Coomassie blue staining (Fig. 1).

Four specific dominant bands in the lane containing immunoprecipitation products from leaf samples infected with BBWV2-53/37-Flag were excised from the gel and subjected to LC-MS/MS analysis. The identified proteins and their MS/MS spectral information are shown in Fig. 1. The bands at positions 320 and 95 kDa were identified as HSP90. While three HSP90 family proteins—HSP90-1, HSP90-2, and HSP90-3—have been characterized in *N. benthamiana* (30), our analysis identified various peptide sequences conserved across the three *N. benthamiana* HSP90 family proteins, as well as a few peptide sequences conserved by HSP90-2 and HSP90-3 but not conserved in HSP90-1 (Fig. S2). In addition, the bands at positions 80 and 45 kDa were identified as ER-luminal binding protein (BiP) and actin, respectively. The identification of HSP90 from the bands at positions 320 and 95 kDa might be attributed to its role as a molecular chaperone that interacts with diverse proteins or its capacity to form oligomers. Based on our approach, we identified three candidate host interacting partners of either VP37 or VP53. In this study, we further investigated whether HSP90 acts as a host factor by interacting with VP37 or VP53 and explored its functional role in BBWV2 infection.

**Fig 2:**
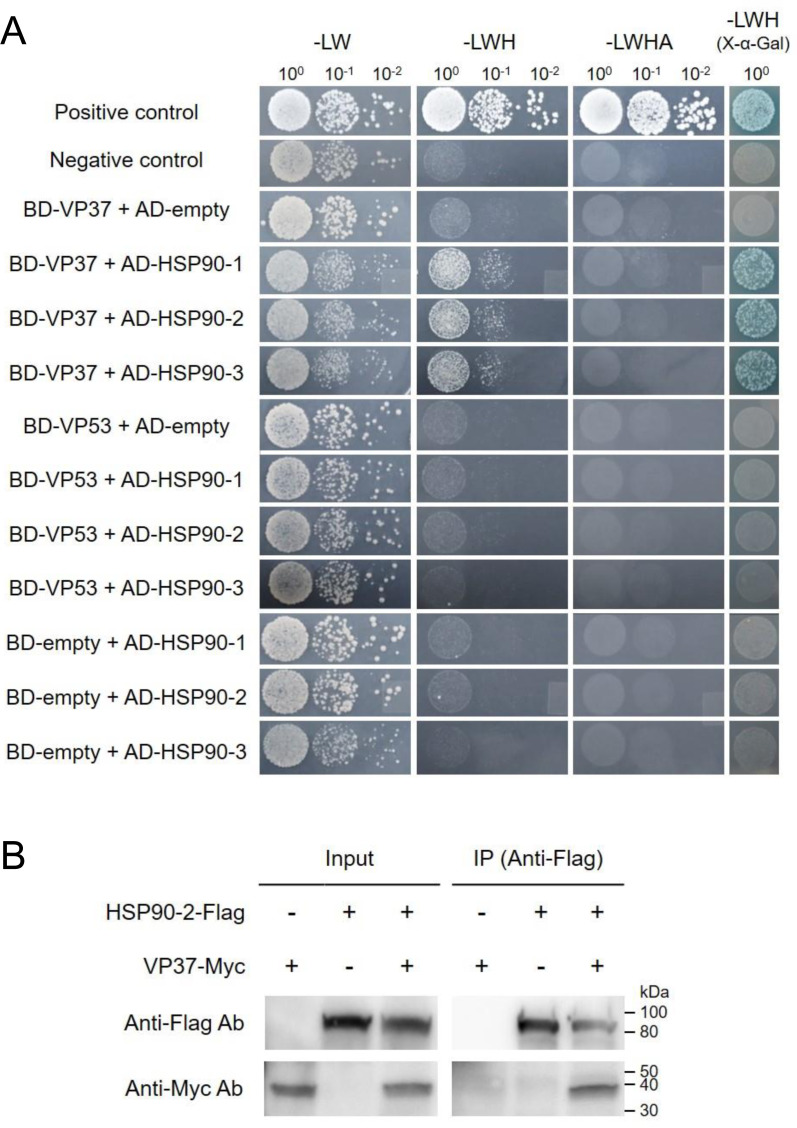
HSP90 is a cellular interacting partner of VP37. (**A**) The interactions between *N. benthamiana* HSP90 family proteins and either VP37 or VP53 in YTHA. VP37 and VP53 were fused downstream of GAL4-BD in pGBKT7, while HSP90-1, HSP90-2, and HSP90-3 were fused downstream of GAL4-AD in pGADT7. Yeast cells co-transformed with the indicated constructs were selected on SD/-LWH and SD/-LWHA agar media following gradient dilution (10^0^, 10^−1^, and 10^−2^), and their α-galactosidase activities were assessed on SD/-LWH agar media containing X-α-Gal. Interactions between the SV40 large T antigen_(84-708)_ and either murine p53_(72-390)_ or human lamin C_(66-230)_ were used as positive and negative controls, respectively. (**B**) Assessment of the HSP90-VP37 interaction through Co-IP. HSP90-2-Flag and VP37-Myc proteins were produced using an *in vitro* coupled transcription/translation system. The HSP90-2-Flag, VP37-Myc, and their combined mixture (HSP90-2-Flag + VP37-Myc) were immunoprecipitated using anti-Flag antibody-conjugated magnetic beads. The resulting products were analyzed by western blotting using anti-Flag or anti-Myc antibodies.

We initially performed YTHA to verify whether *N. benthamiana* HSP90 family proteins interact with VP37 and/or VP53. Our results revealed that VP37 interacts with HSP90-2 and HSP90-3, as well as with HSP90-1 under low stringency conditions (Fig. 2A). However, none of the *N. benthamiana* HSP90 family proteins interacted with VP53 (Fig. 2A). Given that HSP90-2 and HSP90-3 are nearly identical (99.9%) in amino acid sequences and share high amino acid sequence similarity (96.3%) with HSP90-1 (Fig. S2 and S3), we selected HSP90-2 for further analyses.

A Co-IP assay was subsequently performed to confirm the interaction between HSP90-2 and VP37. HSP90-2 and VP37, which were tagged with Flag and Myc, respectively (i.e., HSP90-2-Flag and VP37-Myc), were expressed using an *in vitro* translation system and subjected to Co-IP using anti-Flag antibodies. Our results demonstrated that HSP90-2 directly interacts with VP37 (Fig. 2B; Fig. S4).

**Fig 3:**
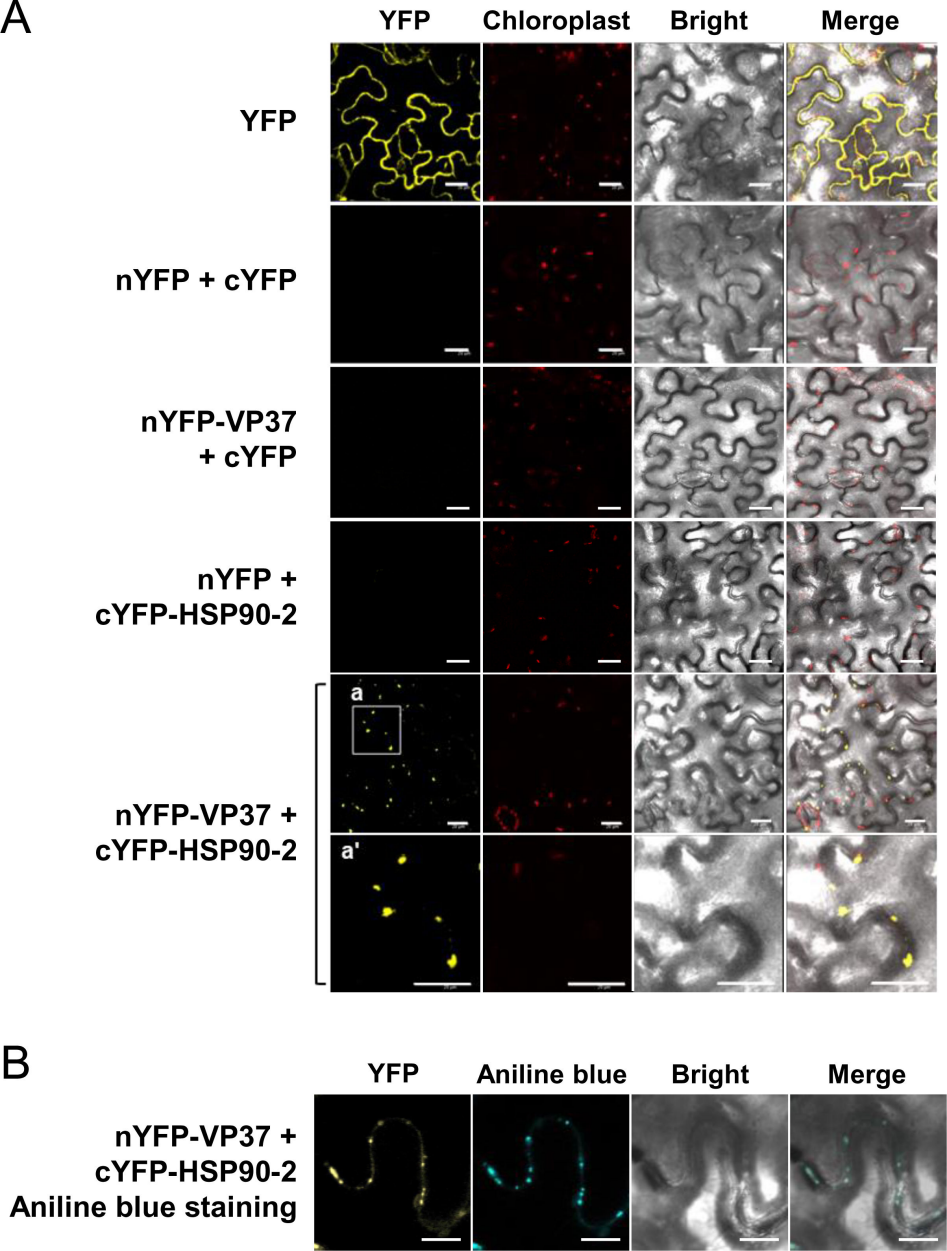
*In vivo* characterization of the HSP90-VP37 interaction using BiFC assay. (**A**) *In vivo* visualization of the HSP90-VP37 interaction. The recombinant proteins indicated on the left side of the panels were expressed in *N. benthamiana* leaves using an *Agrobacterium*-mediated gene expression method. The reconstructed YFP signals in the epidermal cells were observed using confocal microscopy at 3 dpi. Bar = 20 µm. (**B**) The HSP90-VP37 interaction occurs at the PD. The indicated recombinant proteins were expressed in *N. benthamiana* leaves using an *Agrobacterium*-mediated gene expression method. The reconstructed YFP signals in the epidermal cells were observed using confocal microscopy at 3 dpi. Aniline blue-stained callose was served as a PD marker. Bar = 20 µm.

To further confirm the HSP90-VP37 interaction *in planta* and identify the subcellular location of this interaction, we performed BiFC using an *Agrobacterium*-mediated gene expression method (22). VP37 was tagged with nYFP at the N-terminus (nYFP-VP37), whereas HSP90-2 was tagged with cYFP at the N-terminus (cYFP-HSP90-2). nYFP-VP37 and cYFP-HSP90-2 were co-expressed via agroinfiltration in *N. benthamiana* leaves. The reconstructed YFP signals were monitored at 3 dpi in epidermal cells using a confocal microscope. Strong YFP signals were observed as punctate spots along the cell periphery when nYFP-VP37 and cYFP-HSP90-2 were co-expressed (Fig. 3A). These spots were co-localized with aniline blue-stained callose, which served as a PD marker, indicating that HSP90-2 and VP37 interacted at the PD (Fig. 3B). In contrast, consistent with the YTHA result (Fig. 2A), no interaction between nYFP-VP53 and cYFP-HSP90-2 was detected in our BiFC assay (Fig. S5).

### BBWV2 infection upregulates the expression of *HSP90* and induces ER rearrangement in plant cells

**Fig 4:**
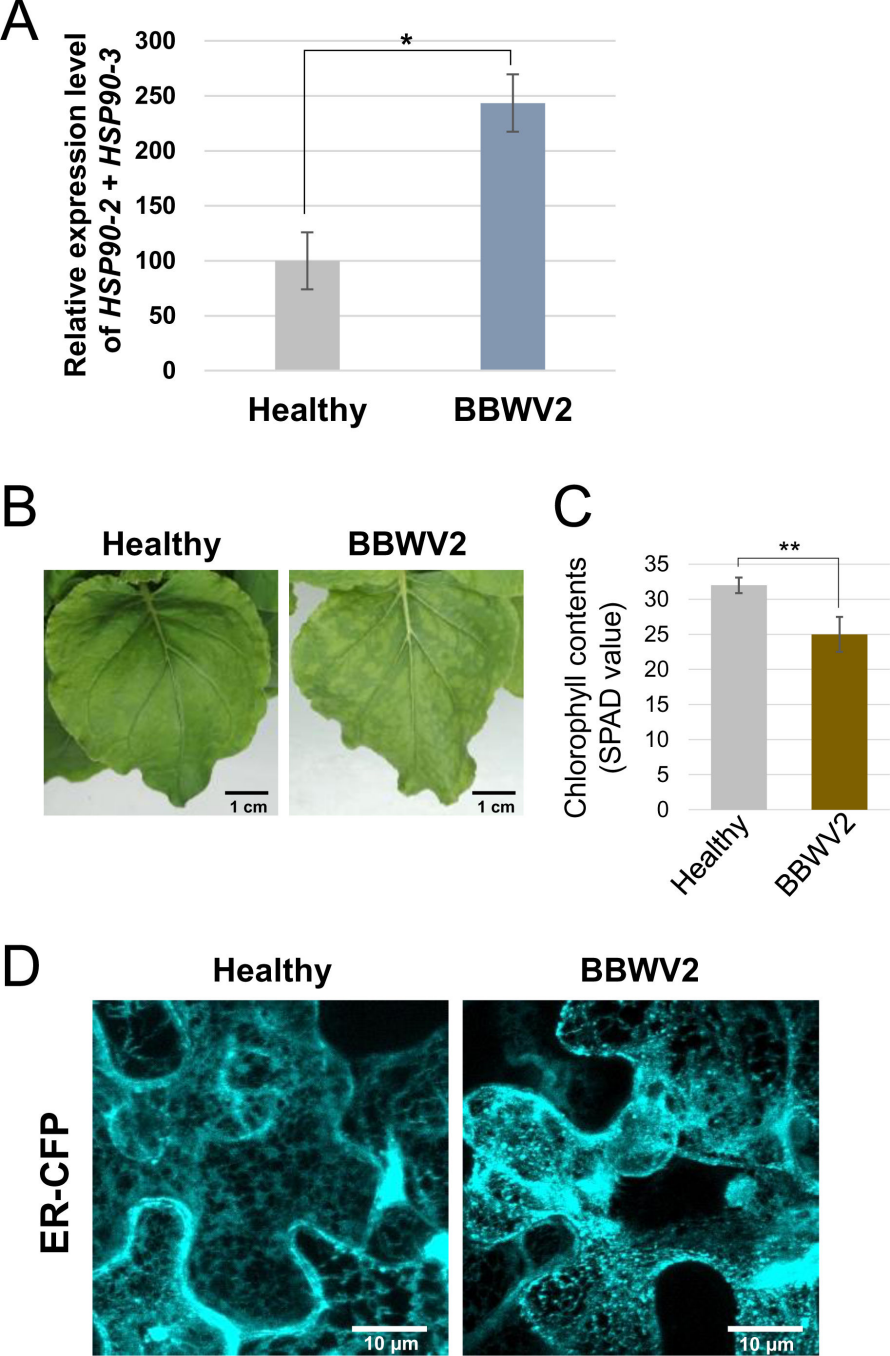
BBWV2 upregulates *HSP90* expression and induces ER rearrangement in infected plant cells. (**A**) BBWV2 infection upregulated the expression of *HSP90-2* and *HSP90-3*. Total RNA was isolated from the upper symptomatic leaves of *N. benthamiana* plants infected with BBWV2 at 12 dpi, and the relative accumulation levels of *HSP90-2* and *HSP90-3* were analyzed by RT-qPCR. Data are presented as the mean ± SD of three replicates with each group containing nine plants. Statistical significance was determined using a paired Student’s *t*-test: *P < 0.05. (**B**) *N. benthamiana* leaves infected with BBWV2 exhibited mosaic symptoms. *N. benthamiana* plants were agroinfiltrated with BBWV2-RP1, and symptoms on the upper uninoculated leaves were observed at 12 dpi. Bar = 1 cm. (**C**) BBWV2 infection caused a decrease in chlorophyll contents. The relative chlorophyll contents in healthy or BBWV2-infected leaves of *N. benthamiana* plants were assessed by measuring soil plant analysis development (SPAD) values. Data are presented as the mean ± SD of three replicates with each group containing nine plants. Statistical significance was determined using a paired Student’s *t*-test: **P < 0.01. (**D**) BBWV2 infection induced ER rearrangement. ER-CFP, an ER marker, was expressed in healthy and BBWV2-infected leaves of *N. benthamiana* plants using an *Agrobacterium*-mediated gene expression method. Subcellular fluorescence signals in the epidermal cells were observed using confocal microscopy at 3 dpi. Bar = 10 µm.

HSP90 is a highly conserved molecular chaperone that plays vital roles in various cellular processes in plants, including protein homeostasis, development, signal transduction, and stress responses (31, 32). HSP90 also plays a multifaceted role in the interaction between plant viruses and their hosts (30, 33, 34). *HSP90* expression is typically upregulated in response to various stresses, including heat, drought, and pathogen attack (32). As shown in Fig. 4A, RT-qPCR analysis revealed that the expression of *HSP90-2* and *HSP90-3* was significantly upregulated upon BBWV2 infection in *N. benthamiana* plants. The upregulation of *HSP90* is strongly associated with ER stress responses (35). In addition, infection with BBWV2 (strain RP1) induced mild mosaic symptoms accompanied by a decrease in chlorophyll content in the plant leaves (Fig. 4B and C). To examine whether BBWV2 alters the ER structure in infected cells, we observed the ER structure after transiently expressing a fluorescent ER marker (ER-CFP) in cells infected with BBWV2. Confocal microscopy revealed that BBWV2 infection induced obvious ER rearrangement, forming punctate bodies along the ER network (Fig. 4D).

### HSP90 is crucial for systemic infection of BBWV2

**Fig 5:**
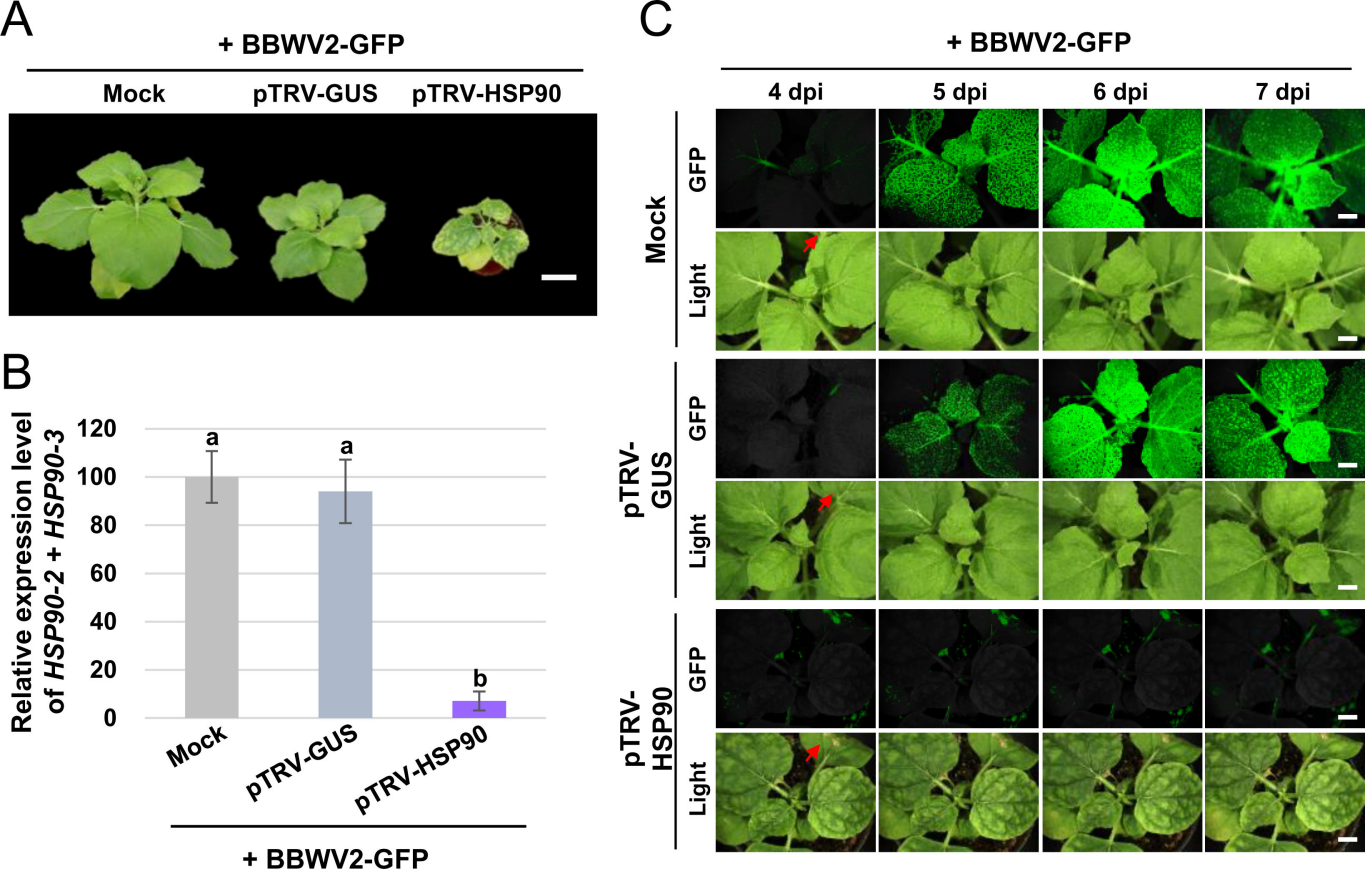
Effects of *HSP90* silencing on the systemic spread of BBWV2. Two-week-old *N. benthamiana* plants agroinfiltrated with mock (*Agrobacterium* containing no binary vector), pTRV-GUS, or pTRV-HSP90. One week later, the plants were agroinfiltrated with BBWV2-GFP. The plants inoculated with mock or pTRV-GUS served as negative controls. (**A**) Phenotypes of BBWV2-infected *N. benthamiana* plants following *HSP90* silencing. *HSP90* silencing caused strong growth inhibition and leaf yellowing in BBWV2-infected *N. benthamiana* plants. The plants were photographed at 14 dpi with BBWV2-GFP. Bar = 5 cm. (**B**) RT-qPCR analysis of the accumulation of *HSP90-2* and *HSP90-3*. Total RNA isolated from the systemic leaves of inoculated *N. benthamiana* plants at 14 dpi was analyzed using RT-qPCR to assess the silencing efficiency of *HSP90-2* and *HSP90-3*. (**C**) Time-course observation of the systemic spread of BBWV2-GFP in *HSP90*-silenced *N. benthamiana* plants. The plants agroinfiltrated with BBWV2-GFP were observed using a FOBI fluorescence imaging system at 4, 5, 6, and 7 dpi. Data shown are representatives of at least three independent experiments. Red arrows indicate the positions of the agroinfiltrated leaves. Bar = 1 cm.

To examine the requirement of HSP90 for BBWV2 infection, we first performed a loss-of-function analysis of *HSP90* using a TRV-based VIGS system (36). *N. benthamiana* plants inoculated with a TRV construct containing a partial sequence of GUS (pTRV-GUS) were used as negative controls (27). Consistent with previous studies (37, 38), *HSP90* silencing resulted in strong growth inhibition and leaf yellowing in *N. benthamiana* plants (Fig. 5A; Fig. S6). To evaluate the silencing efficiency of *HSP90-2* and *HSP90-3*, RT-qPCR was performed using RNA extracted from the systemic leaves of inoculated *N. benthamiana* plants. The result confirmed the efficient silencing of *HSP90-2* and *HSP90-3* (Fig. 5B; Fig. S6). One week after inoculation with the TRV-based VIGS constructs, the plants were agroinfiltrated with BBWV2-GFP (a mixture of *Agrobacterium* cultures containing pBBWV2-RP1-R1 + pBBWV2-R2-GFP), enabling visual tracking of BBWV2 infection dynamics in living plants (18, 25). The inoculated plants were observed using a FOBI fluorescence imaging system starting at 4 dpi with BBWV2-GFP. Although BBWV2-GFP successfully established systemic infection in the plants inoculated with mock or pTRV-GUS, BBWV2 infection was significantly inhibited in *HSP90*-silenced plants (Fig. 5C). Our results suggest that HSP90 is a host cellular protein essential for BBWV2 systemic infection.

### Chaperone function of HSP90 is required for its interaction with VP37 and cell-to-cell movement of BBWV2

**Fig 6:**
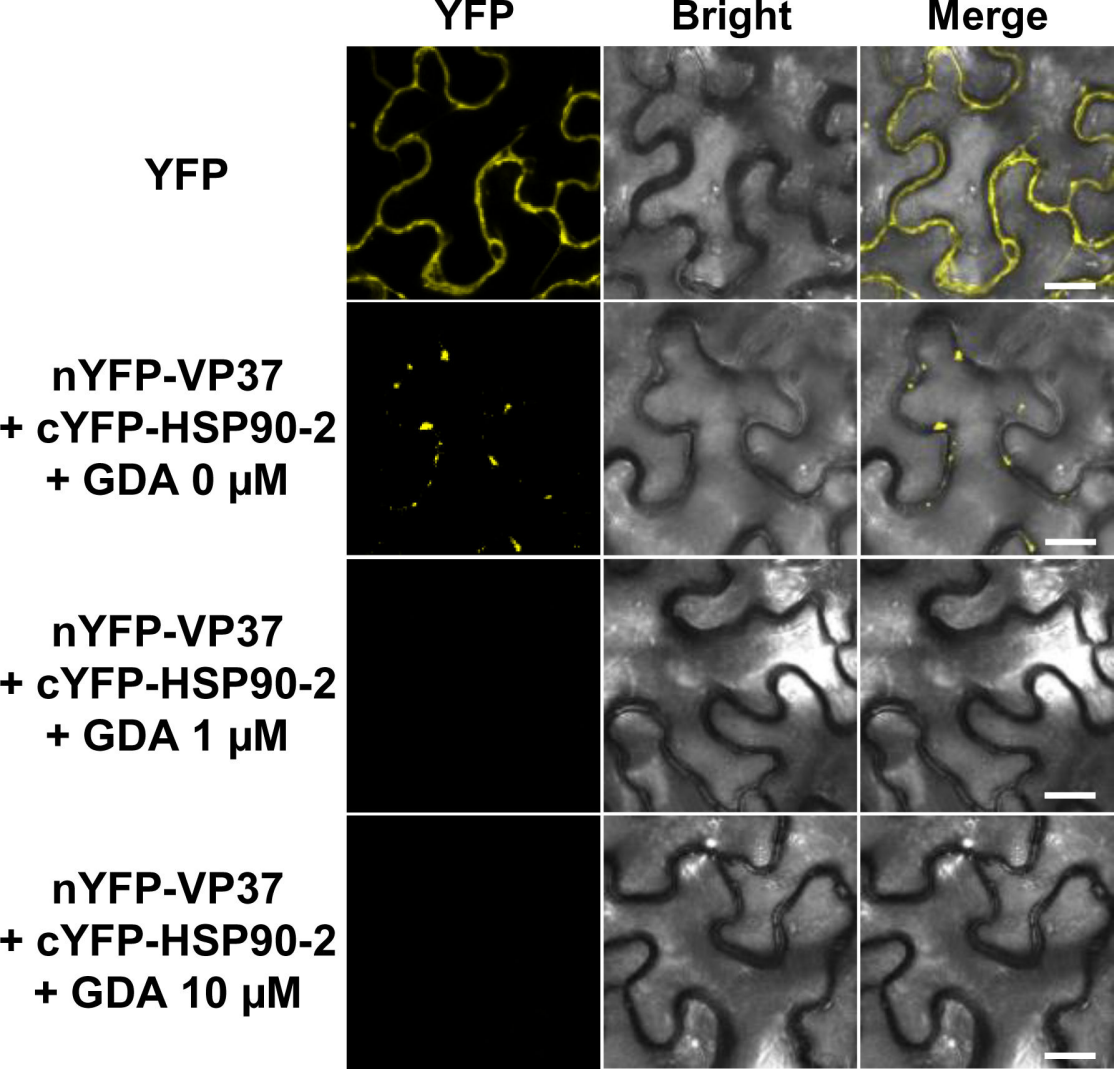
Effects of inhibiting the chaperone function of HSP90 on the HSP90-VP37 interaction. nYFP-VP37 and cYFP-HSP90-2 were co-expressed in *N. benthamiana* leaves using an *Agrobacterium*-mediated gene expression method. After 36 h, 0, 1, or 10 µM geldanamycin (GDA) was syringe-infiltrated into the agroinfiltrated leaf area. The reconstructed YFP signals in the epidermal cells were observed using confocal microscopy at 3 dpi. Bar = 20 µm.

The chaperone function of HSP90 is tightly regulated by the ATPase activity presented by its N-terminal domain (39, 40). The function of HSP90 can be inhibited by GDA, which blocks the ATPase activity of HSP90 (41). To examine whether the chaperone function of HSP90 is required for its interaction with VP37, we performed additional BiFC experiments under GDA treatment conditions. nYFP-VP37 and cYFP-HSP90-2 were co-expressed via agroinfiltration in *N. benthamiana* leaves. After 36 h, the agroinfiltrated leaf areas were syringe-infiltrated with 0, 1, or 10 µM GDA. The interaction between nYFP-VP37 and cYFP-HSP90-2 was then monitored at 3 dpi using confocal microscopy. As shown in Fig. 6, strong YFP signals were observed as punctate spots in the absence of GDA. However, no YFP signals were detected following treatment with 1 or 10 µM GDA (Fig. 6). In addition, GDA treatment did not induce significant changes in the expression or subcellular localization patterns of HSP90 (Fig. S7). These results suggest that the ATP-dependent chaperone function of HSP90 is essential for the HSP90-VP37 interaction.

**Fig 7:**
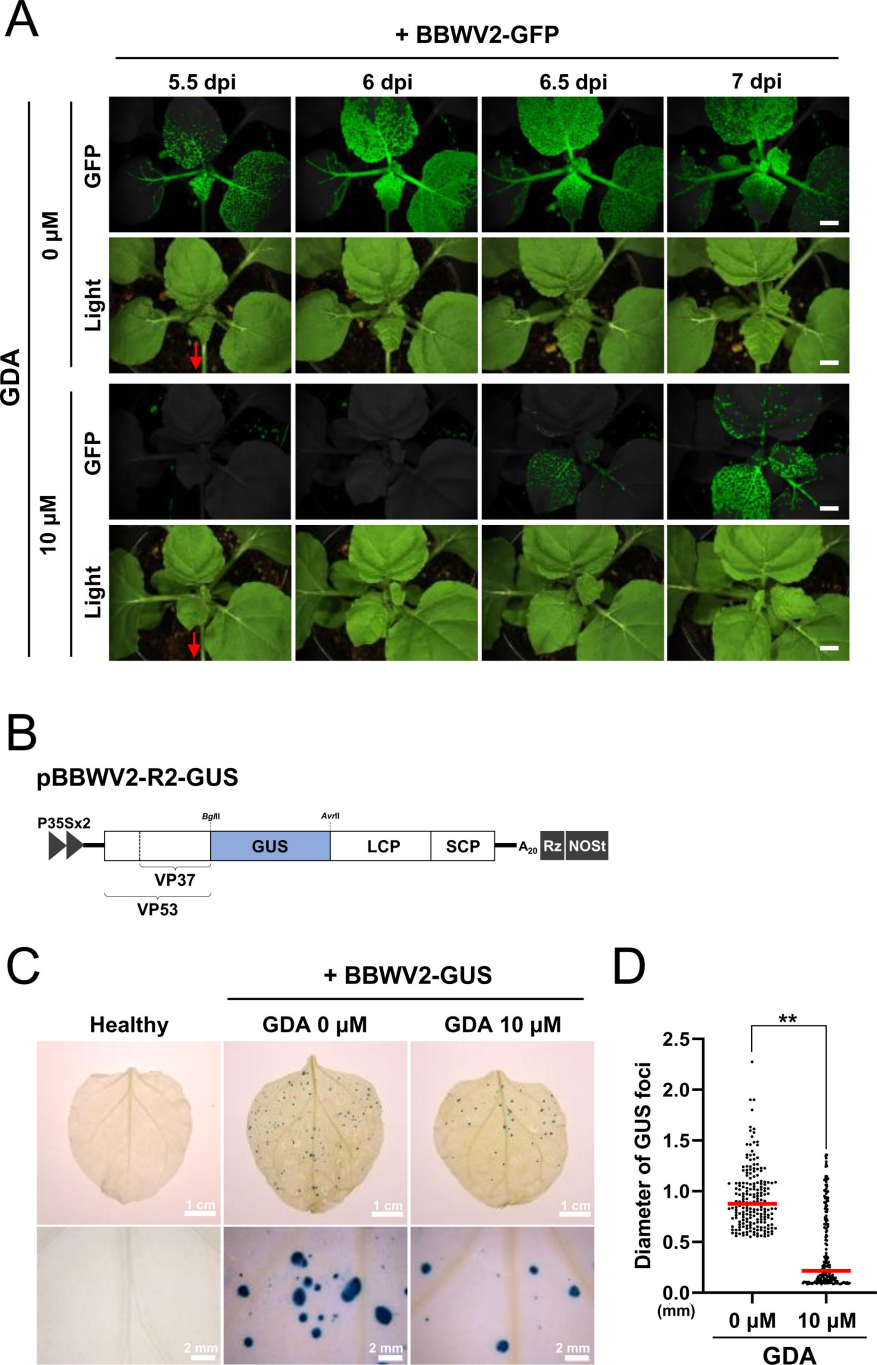
Chaperone function of HSP90 is required for the systemic spread and cell-to-cell movement of BBWV2. (**A**) Effects of inhibiting the chaperone function of HSP90 on the systemic spread of BBWV2. *N. benthamiana* plants agroinfiltrated with BBWV2-GFP. After 36 h, 0 or 10 µM GDA was syringe-infiltrated into the agroinfiltrated leaf area. The plants were observed using a FOBI fluorescence imaging system at 5.5, 6, 6.5, and 7 dpi. Data shown are representatives of at least three independent experiments. Red arrows indicate the positions of the agroinfiltrated leaves. Bar = 1 cm. (**B**) Schematic representation of the BBWV2 RNA2 construct tagged with GUS (pBBWV2-R2-stagRFP), designed to express GUS during viral replication. (**C**) Effects of inhibiting the chaperone function of HSP90 on the cell-to-cell movement of BBWV2. *N. benthamiana* plants were agroinfiltrated with BBWV2-GUS. After 36 h, 0 or 10 µM GDA was syringe-infiltrated into the agroinfiltrated leaf area. The infiltrated leaves were subjected to a histochemical GUS assay at 5 dpi with BBWV2-GUS. The GUS foci were photographed at 5 dpi. Data shown are representatives of at least three independent experiments. (**D**) Statistical analysis of GUS foci diameters shown in panel C. The diameters of the 200 largest GUS foci were measured for each treatment across three independent experiments. The dot plot illustrates the distribution of the GUS foci diameters in ascending order, with the red horizontal bar in each group representing the mean diameter of the measured GUS foci. Statistical significance was determined using a paired Student’s *t*-test: **P < 0.01.

We further examined whether the chaperone function of HSP90 is required for the systemic spread of BBWV2. *N. benthamiana* leaves were agroinfiltrated with BBWV2-GFP. After 36 h, the agroinfiltrated leaf areas were syringe-infiltrated with 0 or 10 µM GDA. The plants were observed using a FOBI fluorescence imaging system starting at 5.5 dpi with BBWV2-GFP. Although strong GFP signals were observed as early as 5.5 dpi in plants treated with 0 µM GDA, clear GFP signals began to appear from 6.5 dpi in those treated with 10 µM GDA (Fig. 7A). This indicated that BBWV2 movement was suppressed in the GDA-treated leaf area, consequently delaying the systemic infection of BBWV2.

We further examined whether the inhibition of HSP90 activity by GDA treatment affects the cell-to-cell movement of BBWV2. *N. benthamiana* leaves were agroinfiltrated with BBWV2-GUS (a mixture of *Agrobacterium* cultures containing pBBWV2-RP1-R1 + pBBWV2-R2-GUS) (Fig. 7B), enabling visual tracking of the cell-to-cell movement of BBWV2. After 36 h, the agroinfiltrated leaf areas were syringe-infiltrated with 0 or 10 µM GDA. At 5 dpi with BBWV2-GUS, the infiltrated leaves were subjected to a histochemical GUS assay to visually measure the extent of viral cell-to-cell movement. BBWV2-GUS spread radially to form infection foci with an average diameter of approximately 930 ± 297.3 µm following treatment with 0 µM GDA (Fig. 7C and D). In contrast, the cell-to-cell movement of BBWV2-GUS was significantly suppressed when treated with 10 µM GDA; the average diameter of foci was approximately 415.2 ± 375.3 µm (Fig. 7C and D). These results suggest that the chaperone function of HSP90 is required for the cell-to-cell movement of BBWV2.

### HSP90-VP37 interaction is required for VP37-derived tubule formation at the PD

**Fig 8:**
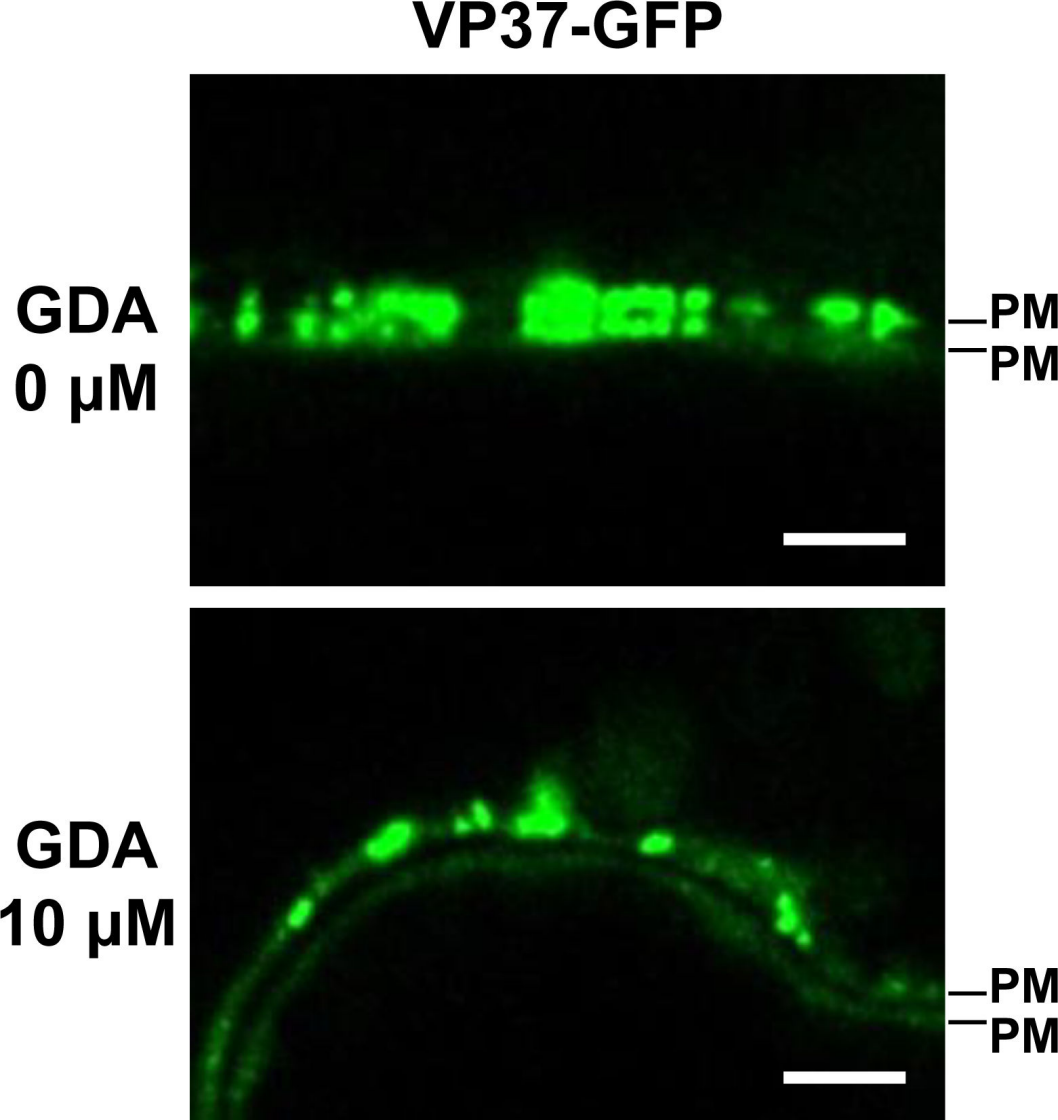
Effects of inhibiting the chaperoning function of HSP90 on VP37-derived tubule formation at PD. VP37-GFP was expressed in *N. benthamiana* leaves using an *Agrobacterium*-mediated gene expression method. After 36 h, 0 or 10 µM GDA was syringe-infiltrated into the agroinfiltrated leaf area. The subcellular localization of VP37-GFP was observed using confocal microscopy at 3 dpi. Data shown are representative of observations from 15 samples across three independent experiments. PM, plasma membrane. Bar = 10 µm.

GFP tagging at the C-terminus of VP37 (VP37-GFP) does not impair its function as a viral movement protein (13, 14). In addition, VP37-GFP is localized to the PD and forms tubule structures when expressed transiently in plant cells (13, 14). As we revealed that the HSP90-VP37 interaction can be inhibited by GDA treatment (Fig. 6), we used GDA treatment to investigate whether this interaction is essential for VP37-derived tubule formation in the PD. VP37-GFP was transiently expressed via agroinfiltration in *N. benthamiana* leaves. After 36 h, the agroinfiltrated leaf areas were syringe-infiltrated with 0 or 10 µM GDA. Next, the subcellular localization of VP37-GFP was observed at 3 dpi using confocal microscopy. In the absence of GDA treatment (0 µM), VP37-GFP predominantly accumulated as punctate spots that were positioned opposite each other on the plasma membranes of two adjacent cells, suggesting the formation of VP37-derived tubules through the PD (Fig. 8). However, when the HSP90-VP37 interaction was inhibited by the treatment with 10 µM GDA, VP53-GFP punctate spots were observed exclusively on the plasma membrane of individual cells, with no corresponding spots on the adjacent cell, suggesting that tubule formation through the PD might be impaired (Fig. 8, the data shown are representative of observations from 15 samples across three independent experiments). Our results suggest that VP37-derived tubule formation through the PD likely requires interaction with HSP90.

## DISCUSSION

During infection, viruses alter the cellular environment of their hosts and utilize various host factors, including cellular proteins (1, 3). In addition, successful systemic infection by plant viruses relies on their ability to move from cell to cell via the PD (8, 9). Interactions between viral and host cellular proteins also play a pivotal role in viral cell-to-cell movement (6). In this study, we identified HSP90 as a host factor that interacts with VP37, the MP of BBWV2. We also demonstrated the importance of this interaction in the cell-to-cell movement and systemic infection of BBWV2.

Although few host proteins have been identified to interact with viral MPs at the PD, pectin methylesterase (PME) is a well-characterized example of a host plasmodesmal protein known to interact with the MPs of tobacco mosaic virus, cauliflower mosaic virus (CaMV), and turnip vein-clearing virus (10, 42, 43). Because PME is primarily present in the cell wall around the PD, it may serve as a general peripheral target for viral MPs in PD localization (42, 44). In addition, the family of PD-located proteins (PDLPs) interacts with MPs of grapevine fanleaf virus (GFLV) and CaMV at the PD, facilitating the movement of these viruses (45). In PDLP knockout plants, tubule formation at the PD and systemic infection with GFLV and CaMV were significantly suppressed (45).

HSP90 is a multifunctional chaperone crucial for protein homeostasis, particularly assisting in the refolding or degradation of misfolded or aggregated proteins that arise from various stress responses (32). HSP90 also plays a critical role in plant-pathogen interactions (46–48). In particular, HSP90 regulates effector-triggered immunity by facilitating the assembly and activation of resistance (R) protein complexes, which involve RAR1 and SGT1 (49, 50). The knockout or silencing of *HSP90* impairs resistance to various pathogens and diminishes the accumulation of R proteins (46–48). In the context of viral infection, HSP90 is involved in the assembly of the replication complexes of various RNA viruses, including bamboo mosaic virus (BaMV) and red clover necrotic mosaic virus (RCNMV) (30, 33). In BaMV, HSP90 facilitates the proper folding of BaMV replicase and recruitment of the viral RNA template during the early stages of replication (30). In RCNMV, HSP90 is essential for viral replication and interacts with p27, a virus-encoded replicase component (33). The infectivity of BaMV and RCNMV was significantly reduced following *HSP90* silencing or GDA treatment in *N. benthamiana* plants (30, 33). However, the role of HSP90 in viral transport remains largely understudied.

In this study, VIGS and GDA treatment experiments demonstrated that HSP90 is required for the systemic spread of BBWV2 (Fig. 5 and 7), indicating that it is a crucial host factor for BBWV2 infectivity. In addition, HSP90-2 interacted with VP37 at the PD (Fig. 3). Interestingly, the formation of VP37-derived tubules through the PD appeared to be impaired when the HSP90-VP37 interaction was inhibited by GDA treatment (Fig. 8). We hypothesized that the inhibition of BBWV2 cell-to-cell movement after GDA treatment may result from impaired tubule formation. These findings emphasize the requirement of the chaperone function of HSP90 in the HSP90-VP37 interaction and BBWV2 cell-to-cell movement, as GDA treatment disrupts the ATP-dependent chaperone activity of HSP90 (41). VP37 forms tubule structures specifically at the PD, but not in the cytoplasm (13, 14, 16). Moreover, the HSP90-VP37 interaction exclusively occurs at the PD (Fig. 3). These observations suggest that the chaperone activity of HSP90 may induce a conformational change in VP37, thereby facilitating the formation of VP37-derived tubules through the PD. Interestingly, a previous study revealed that a significant number of plant viral MPs exhibit unexpected amino acid sequence homology with HSP90 (51). These structural similarities between various plant viral MPs and HSP90 suggest a potential requirement for the functional activities of HSP90, such as its molecular chaperone activity, in facilitating viral cell-to-cell movement. In addition to promoting stable protein folding, molecular chaperones play key roles in the assembly of multimeric protein complexes (52–55). Therefore, it is possible that chaperone activity is involved in the formation of virus-induced intracellular structures, such as tubules, or in the modification of the PD structure to increase its size exclusion limit.

In conclusion, HSP90 specifically interacts with VP37 at the PD, and this interaction is likely essential for the formation of VP37-derived tubules that facilitate viral transport through the PD. Our study further demonstrated that the ATP-dependent chaperone activity of HSP90, which can be disrupted by GDA treatment, is integral to this process, highlighting the important role of molecular chaperones in viral movement. Our findings provide new insights into the molecular mechanisms underlying the cell-to-cell movement of plant viruses.

## Data Availability

The original contributions presented in the study are included in the article/supplemental material; further inquiries can be directed to the corresponding author.

## SUPPLEMENTAL MATERIAL

Supplemental material mbio.02500-24-s0001.pdf Fig. S1-S7 and Table S1.

ASM does not own the copyrights to Supplemental Material that may be linked to, or accessed through, an article. The authors have granted ASM a non-exclusive, world-wide license to publish the Supplemental Material files. Please contact the corresponding author directly for reuse.
