# Supplementary material for: HSP90 interacts with VP37 to facilitate the cell-to-cell movement of broad bean wilt virus 2
Source: mBio. 2025 Feb 19;16(3):e02500-24. doi: 10.1128/mbio.02500-24 (PMC11898612; doi:10.1128/mbio.02500-24)
Supplement: Supplemental material — Fig. S1-S7 and Table S1. [file mbio.02500-24-s0001.pdf]

Supplementary Figure S1

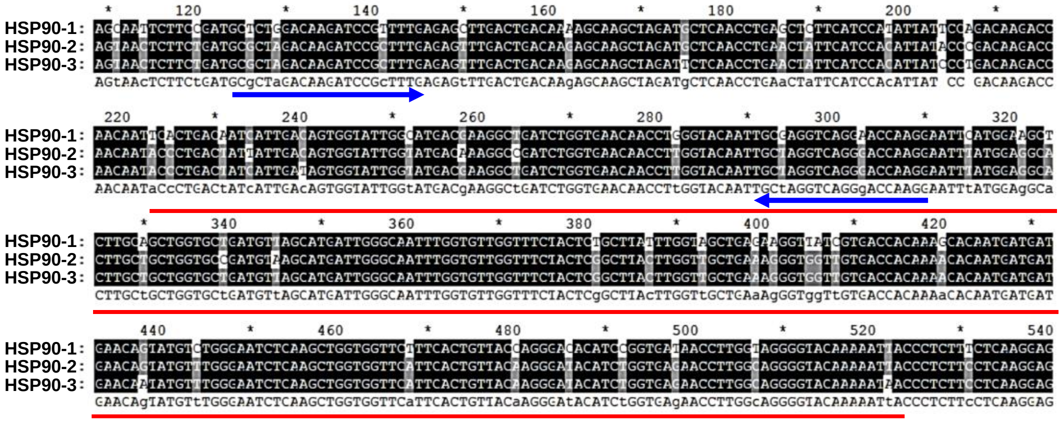

Supplementary Fig. S1. Alignment of the nucleotide sequences of *Nicotiana benthamiana* HSP90 family genes. The positions of primers used for RT-qPCR analysis to quantify the accumulation levels of *HSP90-2* and *HSP90-3* mRNAs are indicated by blue arrows. The partial fragment sequence of *HSP90-2* cloned into pTRV2 is marked with a red line.

Supplementary Figure S2

```
HSP90-1      MADTETFAFQAEINQLLSLIINTFYSNKEIFLRELISNSSDALDKIRFESLTDKSKLDAQ      60
HSP90-2      MAEQETFAFQAEINQLLSLIINTFYSNKEIFLRELISNSSDALDKIRFESLTDKSKLDSQ      60
HSP90-3      MAEQETFAFQAEINQLLSLIINTFYSNKEIFLRELISNSSDALDKIRFESLTDKSKLDSQ      60
             **: ******************************************************:*
                                             RELISNSSDALDKIRFESLTDKS
                                             RELISNSSDALDKIRF       KLDSQ

HSP90-1      PELFIHIIPDKTNNTLTIIDSGIGMTKADLVNNLGTIARSGTKEFMEALAAGADVSMIGQ      120
HSP90-2      PELFIHIIPDKTNNTLTIIDSGIGMTKADLVNNLGTIARSGTKEFMEALAAGADVSMIGQ      120
HSP90-3      PELFIHIIPDKTNNTLTIIDSGIGMTKADLVNNLGTIARSGTKEFMEALAAGADVSMIGQ      120
             ************************************************************
             PELFIHIIPDKT              KADLVNNLGTIARS

HSP90-1      FGVGFYSAYLVAEKVIVTTKHNDDEQYVWESQAGGSFTVTRDTSGENLGRGTKMTLYLKE      180
HSP90-2      FGVGFYSAYLVAERVVVTTKHNDDEQYVWESQAGGSFTVTRDTSGENLGRGTKITLFLKE      180
HSP90-3      FGVGFYSAYLVAERVVVTTKHNDDEQYVWESQAGGSFTVTRDTSGENLGRGTKITLFLKE      180
             *************:*:*************************************:**:***
                                                     RDTSGENLGRG KITKFLKE

HSP90-1      DQLEYLEERRLKDLIKKHSEFISYPISLWVEKTIEKEISDDEDEEEKKDEEGKVEEVDEE      240
HSP90-2      DQLEYLEERRLKDLVKKHSEFISYPISLWVEKTIEKEISDDEDEEEKKDEEGKVEEVDEE      240
HSP90-3      DQLEYLEERRLKDLVKKHSEFISYPISLWVEKTIEKEISDDEDEEEKKDEEGKVEEVDEE      240
             **************:*********************************************
             DQLEYLEERR      KHSEFISYPISLWVEKT
                            KKHSEFISYPISLWVEKT

HSP90-1      KEKEEKKKKKIKEVSNEWSLVNKQKPIWMRKPEEITKEEYAAFYKSLTNDWEEHLAVKHF      300
HSP90-2      KEKEEKKKKKIKEVSNEWSLVNKQKPIWMRKPEEITKEEYAAFYKSLTNDWEEHLAVKHF      300
HSP90-3      KEKEEKKKKKIKEVSNEWSLVNKQKPIWMRKPEEITKEEYAAFYKSLTNDWEEHLAVKHF      300
             ************************************************************
                      KIKEVSNEWSLVNKQ                    KSLTNDWEEHLAVKH
                        KEVSNEWSLVNKQ      KPEEITKEEYAAFYKS           KHF

HSP90-1      SVEGQLEFKAVLFVPKRAPFDLFDTKKKPNNIKLYVRRVFIMDNCEELIPEYLSFVKGIV      360
HSP90-2      SVEGQLEFKAVLFVPKRAPFDLFDTKKKPNNIKLYVRRVFIMDNCEELIPEYLSFVKGIV      360
HSP90-3      SVEGQLEFKAVLFVPKRAPFDLFDTKKKPNNIKLYVRRVFIMDNCEELIPEYLSFVKGIV      360
             ************************************************************
             SVEGQLEFKA     KRAPFDLFDTKK                             KGIV
                     KAVLFVPKR

HSP90-1      DSEDLPLNISREMLQQNKILKVIRKNLVKKCIELFFEIAENKEDFDKFYEAFAKNIKLGI      420
HSP90-2      DSEDLPLNISREMLQQNKILKVIRKNLVKKCIELFFEIAENKEDYDKFYEAFSKNLKLGI      420
HSP90-3      DSEDLPLNISREMLQQNKILKVIRKNLVKKCIELFFEIAENKEDYDKFYEAFSKNLKLGI      420
             ********************************************:*******:**:****
             DSEDLPLNISRE                                            KLGI

HSP90-1      HEDSQNRSKFAELLRYHSTKSGDDMTSLKDYVTRMKEGQNDIYYITGESKKAVENSPFLE      480
HSP90-2      HEDSQNRSKFAELLRYHSTKSGDEMTSLKDYVTRMKEGQNDIYYITGESKKAVENSPFLE      480
HSP90-3      HEDSQNRSKFAELLRYHSTKSGDEMTSLKDYVTRMKEGQNDIYYITGESKKAVENSPFLE      480
             ***********************:************************************
             HEDSQNRS                                         KKAVENSPFLE

HSP90-1      KLKKKGYEVLYMVDAIDEYCIGQLKEFEGKKLVSATKEGLKLDESEDEKKKKEELKEKFE      540
HSP90-2      KLKKKGYEVLYMVDAIDEYSIGQLKEFEGKRLVPTTKEDLKLDESEDEKKKHEELKEKFE      540
HSP90-3      KLKKKGYEVLYMVDAIDEYSIGQLKEFEGKRLVPTTKEDLKLDESEDEKKKHEELKEKFE      540
             *******************.**********:** :***.************:********
             KL

HSP90-1      GLCKVIKDVLGDKVEKVVVSDRVVDSPCCLVTGEYGWTANMERIMKAQALRDSSMAGYMS      600
HSP90-2      GLCKVIKDVLGDKVEKVVVSDRVVDSPCCLVTGEYGWTANMERIMKAQALRDSSMAGYMS      600
HSP90-3      GLCKVIKDVLGDKVEKVVVSDRVVDSPCCLVTGEYGWTANMERIMKAQALRDSSMAGYMS      600
             ************************************************************
                KVIKDVLGDKV

HSP90-1      SKKTMEINPDNAIMDELRKRADADKNDKSVKDLVLLLFETALLTSGFSLDDPNTFGNRIH      660
HSP90-2      SKKTMEINPENAIMEELRKRADADKNDKSVKDLVLLLFETALLTSGFSLDEPNTFGNRIH      660
HSP90-3      SKKTMEINPENAIMEELRKRADADKNDKSVKDLVLLLFETALLTSGFSLDEPNTFGNRIH      660
             *********:****:***********************************:*********

HSP90-1      RMLKLGLSIDEDSGDADVDMPALEDPEA-DAEGSKMEEVD   699
HSP90-2      RMLKLGLSIDEDCGDAEADMPPLEDPEA-DAEGSKMEEVD   699
HSP90-3      RMLKLGLSIDEDCGDAEADMPPLEDTEADDAEGSKMEEVD   700
             ************.***:.*** *** ** ***********
```

Supplementary Fig. S2. Alignment of the amino acid sequences of *Nicotiana benthamiana* HSP90 family proteins and the coverage map of peptides obtained by LC-MS/MS analysis. The gel fragments at positions ~320 and ~95 kDa were excised from the SDS-PAGE gel that separated the immunoprecipitation products derived from leaf samples infected with BBWV2-53/37-Flag (Fig. 1). The excised gel fragments were subjected to in-gel digestion using trypsin followed by LC-MS/MS analysis. The peptide sequences identified by LC-MS/MS analysis are shown below the aligned sequences. The sequences highlighted in red indicate amino acids, which are conserved between HSP90-2 and HSP90-3 but not conserved in HSP90-1.

Supplementary Figure S3

A

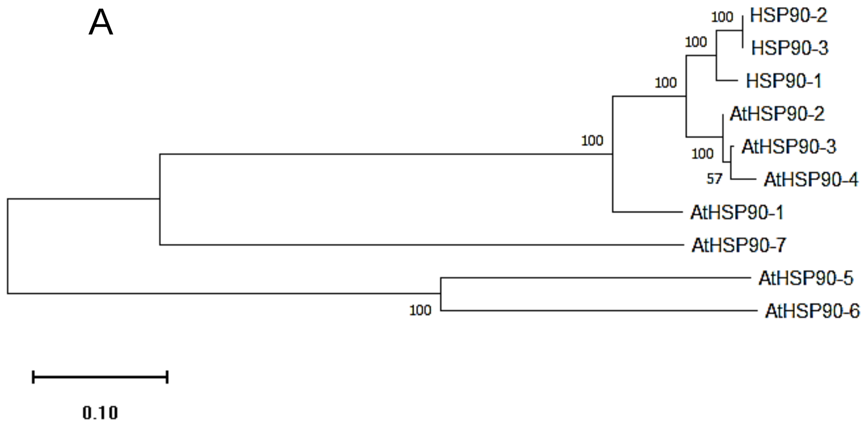

B

	HSP90-2	HSP90-3
HSP90-1	96.4 %	96.3 %
HSP90-2	–	99.9 %

Supplementary Fig. S3. (A) Phylogenetic analysis of HSP90 family proteins from *Nicotiana benthamiana* and *Arabidopsis thaliana*. The analysis was performed using the amino acid sequences by the maximum likelihood method. GenBank accession numbers: HSP90-1 (AY368904), HSP90-2 (AY368905), HSP90-3 (GQ845021), AtHSP90-1 (NP_200076), AtHSP90-2 (NP_001190553), AtHSP90-3 (NP_200412), AtHSP90-4 (NP_200411), AtHSP90-5 (NP_178487), AtHSP90-6 (NP_001319498), and AtHSP90-7 (NP_194150). (B) The amino acid sequence similarity among *N. benthamiana* HSP90 family proteins.

Supplementary Figure S4

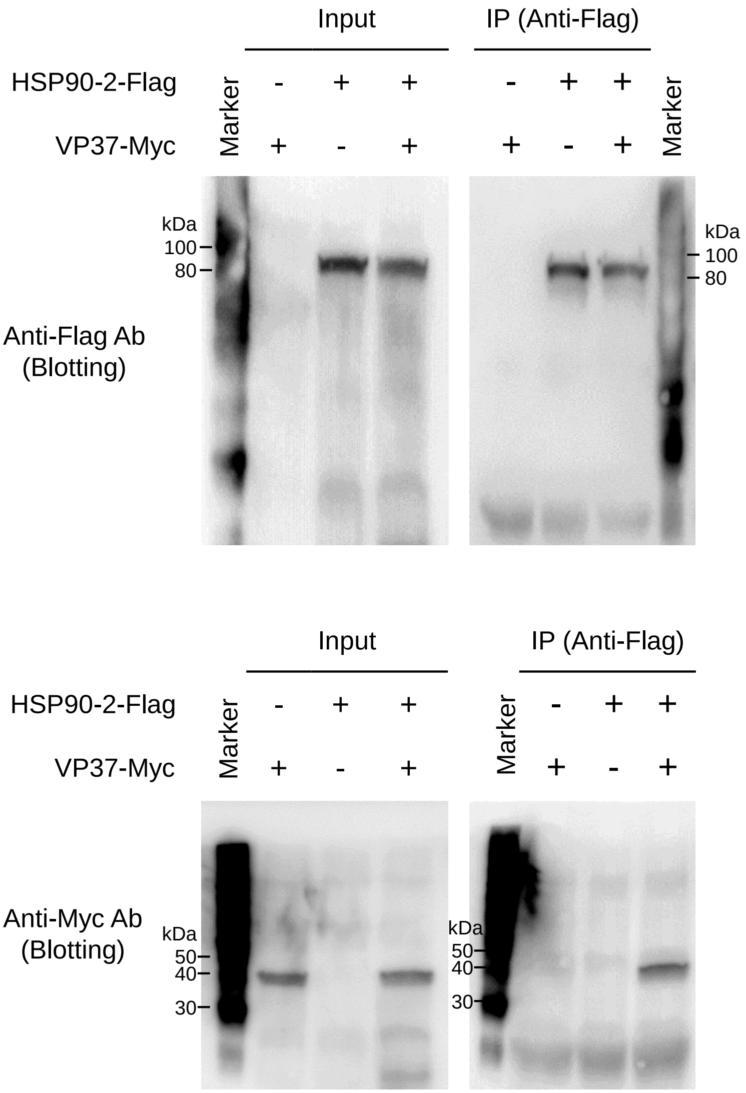

Supplementary Fig. S4. Uncropped blot images for Fig. 2B.

Supplementary Figure S5

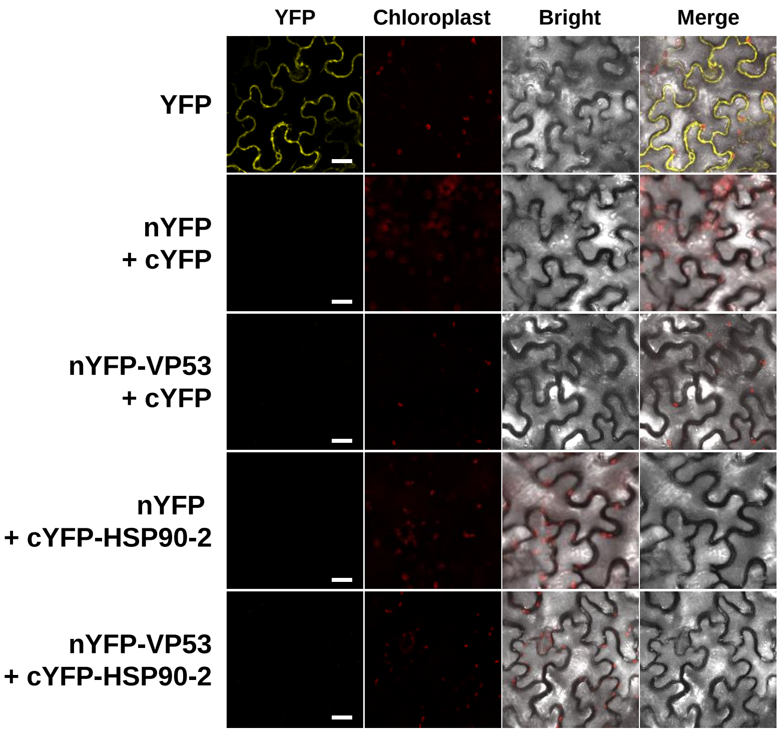

Supplementary Fig. S5. BiFC assay to evaluate the interaction between HSP90-2 and VP53. The recombinant proteins indicated on the left side of the panels were expressed in *Nicotiana benthamiana* leaves using an *Agrobacterium*-mediated gene expression method. Confocal microscopy was used to detect reconstructed YFP signals in the epidermal cells at 3 dpi. Bar = 20 μm.

Supplementary Figure S6

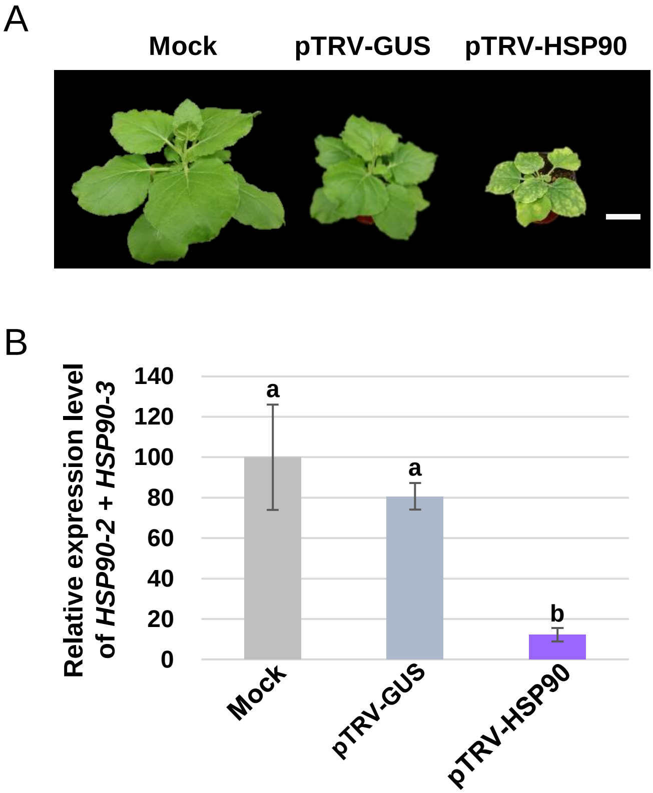

Supplementary Fig. S6. Phenotypes of *HSP90*-silenced *N. benthamiana* plants. Two-week-old *N. benthamiana* plants were agroinfiltrated with mock (*Agrobacterium* containing no binary vector), pTRV-GUS, or pTRV-GUS. The plants inoculated with mock or pTRV-GUS served as negative controls. *HSP90* silencing caused strong growth inhibition and leaf yellowing in *N. benthamiana* plants. The plants were photographed at 21 dpi with the TRV-based VISG constructs. Bar = 5 cm. (B) RT-qPCR analysis of the expression of *HSP90-2 and HSP90-3*. Total RNA isolated from the systemic leaves of *N. benthamiana* plants at 21 dpi was analyzed using RT-qPCR to assess the silencing efficiency of *HSP90-2 and HSP90-3*.

Supplementary Figure S7

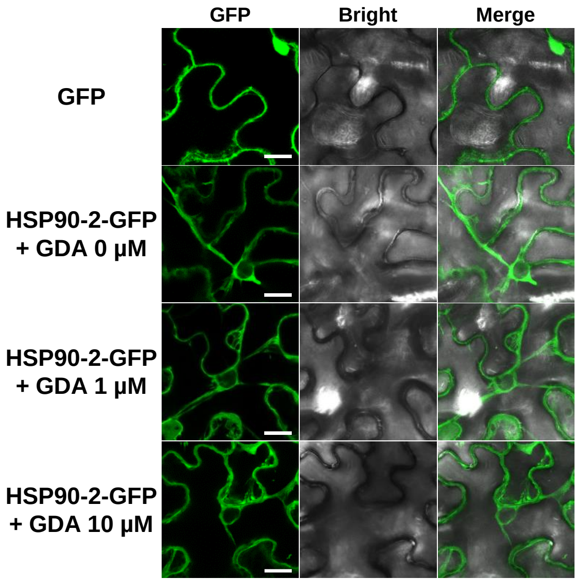

Supplementary Fig. S7. Effects of inhibiting the chaperone function of HSP90 on its subcellular localization. HSP90-2-GFP was expressed in *N. benthamiana* leaves using an *Agrobacterium*-mediated gene expression method. After 36 h, 0, 1, or 10 μM GDA was syringe-infiltrated into the agroinfiltrated leaf area. The subcellular localization of HSP90-2-GFP in the epidermal cells was observed using confocal microscopy at 3 dpi. Bar = 20 μm.

Supplementary Table S1. Primers used in this study.

Primer	Primer sequence (5' to 3')	Purpose
VP37-EcoRI-Fw	ACGAATTCATGAATGAGGCAAATATCAC	To construct pGBKT7-VP37
VP53/37-BamHI-Stop-Rv	CGGGATCCTATTGACCATATCTATAATC	
VP53-EcoRI-Fw	ACGAATTCATGCGTCCCGAACTTGTTG	To construct pGBKT7-VP53
VP53/37-BamHI-Stop-Rv	ACAAGTTCGGGACGTTATACAGCATGTTCA	
HSP90-Fw2	GATGGCGGAGGCAGAGACGTT	To construct pGADT7-HSP90-1, pGADT7-HSP90-2, and pGADT7-HSP90-3
HSP90-Rv2	CCGCTCGAGTTAGTCAACTTCCTCCATCTTGCT	
HSP90-T7-Fw	TTGAGATCTTTAATACGACTCACTATA	To amplify HSP90-2-Flag templates for *in vitro* coupled transcription/translation assays
3E-PolyA-Rv	TTTTTTTTTTTTTTTTTTTTTTTTTTTTTTTTGGGTTATGCTA GTTATGCGG	
VP37-T7-Fw	TTGGAATTTGTAATACGACTCACTATA	To amplify VP37-Myc templates for *in vitro* coupled transcription/translation assays
3E-PolyA-Rv	TTTTTTTTTTTTTTTTTTTTTTTTTTTTTTTTGGGTTATGCTA GTTATGCGG	
VP37-SalI-1-Fw	ACGCGTCGACATGAATGAGGCAAATATCAC	To construct pENTR™1A-VP37
VP37/53-XhoI-1014-Rv	CCGCTCGAGGGTTATTGACCATATCTATAATC	
VP53-SalI-1-Fw	ACGCGTCGACATGCGTCCCGAACTTGTTGC	To construct pENTR™1A-VP53
VP37/53-XhoI-1014-Rv	CCGCTCGAGGGTTATTGACCATATCTATAATC	
HSP90-SalI-1-Fw	ACGCGTCGACATGGCGGAGGCAGAGACG	To construct pENTR™1A-HSP90-2
HSP90-Rv3	ACTCGCTCGAGTTARTCAACTTCCTCCATCTTGCT	
HSP90-Fw1	ATGGCGGAGGCAGAGACGTT	To construct PZP-HSP90-2-GFP
HSP90-SpeI-Rv	AGGACTAGTTTAGTCAACTTCCTCCATCTTG	
GUS-1-BglII-Fw	GAAGATCTATGTTACGTCCTGTAGAAACCCCA	To construct pBBWV2-R2-GUS
GUS-1809-AvrII-Rv	GATCCTAGGTTGTTTGCCTCCCTGCTGCGG	
HSP90-Vigs-SalI-223-Fw	TACGCGTCGACACCCTGACTATTATTGACAGTGG	To construct pTRV2-HSP90
HSP90-Vigs-SalI-523-Rv	TACGCGTCGACTAATTTTTGTACCCCTGCCAAGGTTC	
